# The Effect of Ouhyul Herbal Acupuncture Point Injections on Shoulder Pain after Stroke

**DOI:** 10.1155/2013/504686

**Published:** 2013-06-13

**Authors:** Yu-Ri Seo, Woo-Sang Jung, Seong-Uk Park, Sang-Kwan Moon, Jung-Mi Park, Joo-Young Park

**Affiliations:** ^1^Department of Cardiovascular & Neurologic Diseases, College of Korean Medicine, Kyung-Hee University, Seoul, Republic of Korea; ^2^Department of Cardiovascular and Neurologic Diseases, Hospital of Oriental Medicine, Kyung Hee University, 1 Hoegi, Seoul 130-702, Republic of Korea

## Abstract

An effective and safe remedy for shoulder pain is needed as shoulder pain is a common complication of stroke and restricts recovery of patients. This study was carried out to evaluate the effect of Ouhyul herbal acupuncture point injection (O-API) on shoulder pain in patients with stroke. Twenty-four participants with shoulder pain after stroke were recruited and randomized to the O-API and control groups. Treatment was conducted for 2 weeks three times per week. We evaluated the effects of treatment with a numerical rating scale (NRS), painless passive range of motion (PROM) of external shoulder rotation, and the Fugl-Meyer Motor Assessment (FMMA) at baseline, each week, and 1 week after the final treatment. All measures were similar between the O-API and control groups at baseline. The O-API group showed significant improvement on the NRS compared with that in the control group after 2 weeks of treatment, and the treatment effect was maintained until the follow-up period. PROM decreased significantly in both groups, but the reduction was maintained only in the O-API group. No significant difference was observed on the FMMA between the two groups. O-API resulted in significant improvement in shoulder pain after stroke, and its effect was maintained after termination of treatment without any severe side effects.

## 1. Introduction

Shoulder pain is one of the most common stroke complications (9–84%) [1, 2]. Various controversial theories about the mechanisms generating shoulder pain after stroke have been proposed, but the most plausible theories attribute the cause to subluxation, which occurs easily after stroke because of the structural characteristics of the shoulder, spasticity, and contracture that originate as a change in muscle balance after an upper motor neuron disorder. Rotator cuff abnormalities and flaccid muscles near the glenohumeral joint are prone to trauma [3].

Posstroke shoulder pain is generally treated with nonsteroidal anti-inflammatory drugs, steroid injections, positioning and handling, shoulder strapping, or electrical stimulation, but these are imperfect methods for treating shoulder pain after stroke because of their side effects, insufficient effect duration, and statistically insignificant effects [4].

Patients with stroke and severe shoulder pain are apt to withdraw from rehabilitation programs [5], stay longer in the hospital [6], and complain of a poor quality of life [7]; thus, an effective remedy for reducing shoulder pain after stroke is strongly needed to promote rehabilitation treatment. Ouhyul herbal acupuncture point injection (O-API) is a treatment used in Korean medical hospitals, in which a herbal drug-like plant extract is injected at acupuncture points.

Therefore, in this study, we evaluated the effect of O-API on shoulder pain after stroke by measuring pain relief, functional improvement in the shoulder joint, and the increase in shoulder range of motion.

## 2. Materials and Methods

### 2.1. Study Design

This was a prospective, randomized, double-blind, and controlled clinical trial conducted from September 2010 to February 2012 at the Kyung-Hee Korean Medical Center, Korea. 

Participants were randomly allocated to the O-API or the normal saline- (NS-) API groups. They were randomized off-site using a blocked stratified procedure that consisted of a block size of four with one stratum and two groups with different baseline NRS scores (>5 and ≤5). The randomization was conducted by an independent physician who was not involved in the inclusion or exclusion process, treatment, or assessment procedures. We use the result of the study about the effect of Bee venom acupuncture point injection on the shoulder pain after stroke as a significant level of more than 95% and power of 80%, and we decided to recruit 30 patients for the study.

Participants were screened for eligibility using a chart review and interview on the first visit and randomly assigned to the groups. They were treated by the O-API or the NS-API three times per week during the 2-week treatment. Effectiveness was assessed every week, and participants received at least four treatments. Participants attended a follow-up session 1 week after the final treatment and were assessed. 

### 2.2. Study Participants

The study participants were recruited by referral from Korean medical doctors at Kyung-Hee Korean Medical Center. Patients were recruited through the advertisement. 

Participants were informed about the study protocol and allowed to be included in this study after their written consent. This study protocol was approved by the Institutional Review Board of Kyung-Hee Korean Medical Center (KOMC IRB 2010-10).

Inclusion criteria were (1) shoulder pain with a NRS > 2, (2) alert mental status and ability to answer the survey, (3) no treatment plans that would affect shoulder pain during the study, and (4) consent to join the study. 

Exclusion criteria were (1) patients with any infection, abscess, and those who were unable to receive invasive treatment due to unstable vital signs, (2) a history of fracture or trauma at the shoulder prior to stroke onset, and (3) patients who complained of shoulder pain as a result of fracture or trauma after stroke. 

And we recruited patients without division into acute or subacute.

### 2.3. Intervention

The O-API was prepared at Kyung-Hee Korean Medical Center and the NS-API was produced at JW Pharmaceutical, a Korean drug manufacturing company. O-API consists of eight herbal medicines, including 20 g Gardeniae Fructus, 8 g Corydalis Tuber, 8 g olibanum, 8 g myrrha, 6 g Persicae Semen, 6 g Paeoniae Radix Rubra, 6 g Salviae Miltiorrhizae Radix, and 4 g Sappan Lignum. The herbs were placed in 660 mL distilled water and refluxed for 2 hours, and the liquefied steam was collected in a flask. The mixture was brought up to 200 mL with distilled water, and 1.8 g NaCl was added. The solution was filtered and subdivided into 20 mL sterilized bottles, closed with an aluminum cap, and sterilized in an autoclave. There are studies about herbal acupuncture point injection that herbal acupuncture point injection has an effect on headache, cervical spondylosis, and knee osteoarthritis.

We followed the STRICTA guidelines. Korean API is based on the modern innovation of traditional acupuncture and strengthens and sustains the effects of acupuncture. In this study, five needles were inserted per participant per session and unilateral LI 15, TE 14, GB 21, SI 11, and SI 12 were used as acupuncture points. Needles were applied to the subcutaneous tissue at the acupoints. No muscle twitch response was observed, but participants felt a needling sensation as the fluid was injected. After injecting 0.1 cc at each point, the 30-gauge needle was pulled out immediately. These processes were also adjusted to normal saline group. Participants received two treatment sessions and were observed at one follow-up session. Each session was provided after a 1-week interval, and participants received three treatments per session. Participants could receive any other treatment but they should not change treatments likely to affect their shoulder pain from 1 week before the start of our study to the follow-up session. The practitioner in this study had 6 years of education and 2 years of clinical experience working at Kyung-Hee Korean Medical Center.

A placebo effect may be associated with pain control, but the NS injection only had sodium chloride as the control.

### 2.4. Outcome Measures

Participants were evaluated by the numerical rating scale (NRS), painless passive range of motion (PROM) of external shoulder rotation, and the Fugl-Meyer Motor Assessment (FMMA) and the McGill Pain Questionnaire-short form (MPQ-SF) at each visit by the same examiner blinded to group allocation. The NRS score revealed subjective pain intensity level from 0 (no pain) to 10 (the most intense pain imaginable). 

The MPQ-SF is a multidescriptive measure of pain that consists of 22 pain questions. It reveals the nature of shoulder pain. 

PROM is the painless passive range of motion of shoulder external rotation. Participants were placed in a supine position with 45° abduction of the shoulder joint and 90° flexion of the elbow joint with the forearm pronated. The examiner externally rotated the shoulder until the subjects felt pain and measured the angle between the line connecting the olecranon to the styloid process of the ulna and the horizontal plane. PROM is an objective pain level measure. We assessed PROM with goniometer after treatment in each session. The normal range of PROM is 0–90 degrees. And PROM decreases as symptoms are improved.

FMMA is a scale that shows motor function of the upper extremities and consists of assessment items segmented for several extremity regions. Unlike other assessment scales, it reveals shoulder function. 

Detailed information on these measurement items and relevant scores are listed in the Appendix (see Supplementary Material available online at http://dx.doi.org/10.1155/2013/504686). 

### 2.5. Statistical Analysis

The analysis was based on a per-protocol basis. Between-group differences in demographic and baseline characters were tested with the chi-square test for categorical variables and the Mann-Whitney *U*-test for continuous variables. Although this trial's sample size was small, we confirmed that our results were normally distributed using the Kolmogorov-Smirnov test. We used the paired *t*-test for within-group comparisons and the independent *t*-test for between-group comparisons and to check for changes in each outcome measure. All results are expressed as mean ± standard deviation. SPSS 12.0 for Windows was used for the analyses (SPSS, Inc., Chicago, IL, USA). A *P* < 0.05 was considered significant.

## 3. Results

### 3.1. Baseline Participant Characteristics

Twenty-nine participants were included. Three participants in the O-API group and two in the NS-API group withdrew or were lost to followup at 5 days after the endpoint of the study. Thirteen participants in the O-API group and 11 in the NS-API group completed the interventions and provided complete data at the followup. Therefore, 24 participants were included in the analysis.  Table 1 shows the baseline characteristics of the study population. No differences were observed in the baseline characteristics between the groups.

The participants usually described their pain as “heavy, splitting, fearful, or hot-burning” on the MPQ-SF. 

### 3.2. Changes in NRS Scores

NRS scores were not significantly different between the groups at baseline. A significant decrease in NRS score was observed in the O-API group after treatment but not in the NS-API group (*P* = 0.267). The significant difference in the NRS score in the O-API group (*P* = 0.000) was maintained at the 1-week followup, whereas NRS score in the NS-API group showed no decrease compared with that at baseline. 

**Figure 1:**
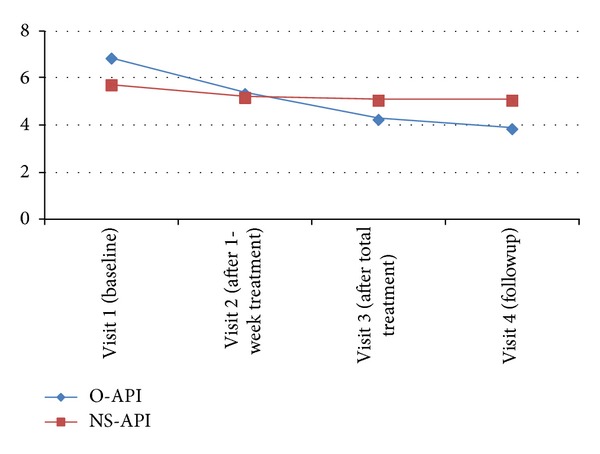
Numerical rating scale (NRS) scores in each group during the trial.

The O-API group demonstrated a significant change according to the independent *t*-test compared with that in the NS-API group (Figure 1).

### 3.3. Changes in PROM Scores

The PROM score in the O-API group was similar (48.85 ± 21.03) to that in the NS-API group (33.55 ± 19.43) prior to treatment. Significant decreases in PROM scores were observed compared with those at baseline in both groups. PROM score decreased after treatment in the O-API group (12.23 ± 10.96) but was not different from that in the NS-API group (5.36 ± 7.66). 

**Figure 2:**
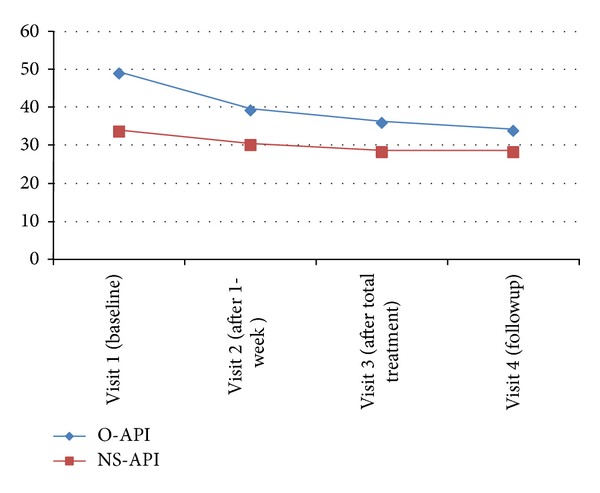
Passive range of motion (PROM) of external shoulder rotation in each group.

However, the decrease in PROM in the O-API group from baseline to the 1-week follow-up period was significant, whereas that in the NS-API group was not (Figure 2). 

### 3.4. Changes in FMMA Scores

Baseline FMMA scores were not different between the two groups. FMMA scores in both groups increased significantly after treatment, and the increases were significantly different between the two groups. The O-API group (1.92 ± 1.19) demonstrated greater improvement than that in the NS-API group (0.91 ± 1.04) (*P* = 0.039).

**Figure 3:**
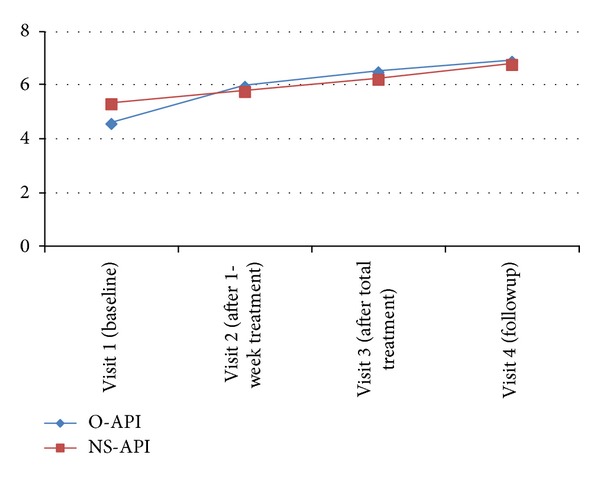
Fugl-Meyer Motor Assessment (FMMA) in each group.

 The increases on the FMMA in the two groups from baseline to the 1-week follow-up period were significantly sustained (Figure 3). 

### 3.5. Adverse Events

One of the 16 O-API participants complained of pantalgia during the study period. Among 13 subjects, one in the NS-API group had transient local site pain and one complained of fatigue. 

No serious adverse events were reported. 

We assessed the adverse effect with the subjective and objective symptoms that participants complain. And we considered of them in a mild level when patients do not need the treatment, a moderate level when patients need the treatment without dropout, and a severe level when patients need treatment with drop out.

## 4. Discussion

This study was a well-designed randomized clinical trial to evaluate the effects of O-API to treat shoulder pain after stroke. As a result, we confirmed that O-API not only reduced shoulder pain after stroke but also rehabilitated functional disorders of a hemiplegic extremity based on the results of the NRS and FMMA. 

The O-API group showed significant improvement on then NRS compared with that in the NS-API group and the improvement was maintained until the follow-up period (1 week after the end of the study).

FMMA and PROM showed significant improvements in both groups at the final treatment session. A significant effect was detected in the PROM scale of the O-API group compared with that in the control group, indicating that both O-API and NS-API had an effect on shoulder range of motion and function of painful shoulders but that O-API showed more improvement in functional recovery than that of NS-API. 

When we checked the scale at 1 week after the end of the study, the PROM effect in the O-API group was maintained at the followup but both treatments had a similar significant effect on the FMMA. This result indicates that O-API has a continuous effect on PROM compared with that of NS-API but that both NS-API and O-API resulted in continuous improvement in shoulder joint function. 

O-API had a better effect on subjective pain sensation than that of NS-API and its effects were maintained. O-API and NS-API both improved limits of shoulder range of motion, but O-API had a more lasting effect. O-API has more significant improvement than that of NS-API in shoulder joint function but only during the treatment period. 

We chose O-API as an alternative treatment because of its pain control effect [8], but no experimental studies are available about how O-API improves shoulder pain after stroke. Some clinical trials have shown that O-API has a significant analgesic effect on somatic pain such that from whiplash injury [9] and low back pain [10]. Several studies have reported the effects of O-API in rats. For example, acupuncture point injection of olibanum (one of the O-API components) has an analgesic effect by changing the amount of serotonin [11]. Cho et al. reported that when a Gardeniae Fructus aqueous extract is injected into the sprained ankle of rats, it improves stepping force of the ankle-sprained limb and decreases paw edema produced by ankle sprain. These analgesic effects on ankle sprain pain can be explained due to the regulation of nitric oxide by suppressing inducible nitric oxide synthase and cyclooxygenase-2 (COX-2) protein expression [12].

Furthermore, a report about the analgesic effect of an acetic acid Persicae Semen extract on mice exposed to a hot plate has been published [13].

In addition, studies about the oral administration of each herbal medicine in the Ouhyul herbal acupuncture mixture reveal a pain-controlling effect. A *Salvia miltiorrhiza* extract decreases tumor necrosis factor-*α* and COX-2 mRNA *in vitro* [14]. Paeoniae Radix improved pain in an *in vivo* experiment that used the stretching and Randall-Selitto methods [15].

Some limitations of this study should be mentioned. The follow-up duration was too short, and it was unclear how to treat the shoulder pain with O-API after stroke. Experimental studies about which herbs in O-API are responsible for the shoulder pain effects are needed. Regardless of these limitations, this trial was important because it evaluated shoulder joint function and the pain and duration of the effect unlike previous O-API studies. 

## 5. Conclusion

The results showed that the O-API was effective for shoulder pain after stroke without critical side effects. Sustainability of the treatment effect was also demonstrated in this trial; thus, we conclude that O-API is a suitable therapy for shoulder pain. A larger, longer term follow-up study is needed to confirm the results. 

## Supplementary Material

Information on the measurementitems and relevant scores. Participants were evaluated by thenumerical rating scale (NRS), painless passive range ofmotion (PROM) of external shoulder rotation, and the Fugl-Meyer Motor Assessment (FMMA), and the McGill Pain Questionnaire-short form (MPQ-SF) at each visit by the same examiner blinded to group allocation. The NRS scorerevealed subjective pain intensity level from 0 (no pain) to 10 (the most intense pain imaginable).

## Figures and Tables

**Table 1:** Baseline demographic and clinical characteristics of the participants.

Characteristic	O-API	NS-API	*P* value
Age (years)	63.8 ± 10.8	67.4 ± 7.8	0.416
Sex (male : female)	6 : 7	4 : 7	0.697
Duration of disease (day)	56.5 ± 29.7	53.5 ± 26.7	1.000
Number of treatment times	5.7 ± 0.7	5.6 ± 0.8	0.858
The other treatment for shoulder pain after stroke (no.)	3	1	0.596
Western medicine	1	1	1.000
Herbal medicine	0	0	1.000
Physical treatment	2	0	1.000
Acupuncture and moxibustion	0	0	1.000
Stroke type (infarction : hemorrhage)	11 : 2	5 : 6	0.082
Stroke recurrent (no.)	1	1	1.000
Underlying disease (no.)			
Hypertension	8	11	0.041
Diabetes	4	3	1.000
Dislipidemia	7	2	0.105
Heart disease	1	2	0.576
