# Supplementary material for: The Effect of Ouhyul Herbal Acupuncture Point Injections on Shoulder Pain after Stroke
Source: Evid Based Complement Alternat Med. 2013 Jun 13;2013:504686. doi: 10.1155/2013/504686 (PMC3697148; doi:10.1155/2013/504686)
Supplement: Supplementary file 1 — Information on the measurementitems and relevant scores. Participants were evaluated by thenumerical rating scale (NRS), painless passive range ofmotion (PROM) of external shoulder rotation, and the Fugl-Meyer Motor Assessment (FMMA), and the McGill Pain Questionnaire-short form (MPQ-SF) at each visit by the same examiner blinded to group allocation. The NRS scorerevealed subjective pain intensity level from 0 (no pain) to 10 (the most intense pain imaginable). [file 504686.f1.docx]

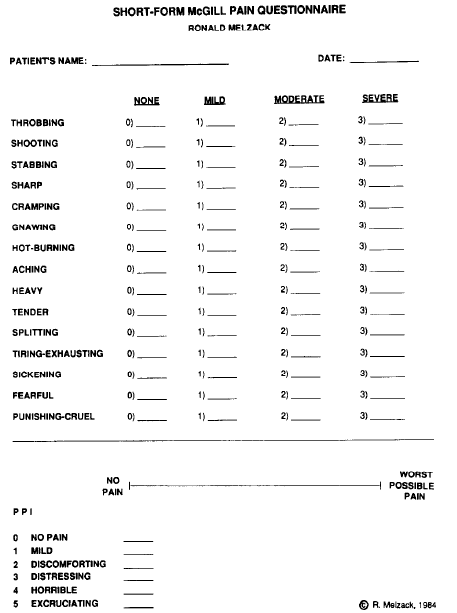


**Appendix**

**Appendix**

Fugl-Meyer Assessment – Motor Domain Items

(shoulder part)

Shoulder retraction

Shoulder elevation

Shoulder abduction

Shoulder abduction to 90 degrees

Shoulder adduction/internal rotation

Shoulder external rotation

Shoulder flexion 0-90 degrees

Shoulder flexion 90-180 degrees
